# Efforts to evaluate translational science’s impact on biomedicine and society should incorporate Science and Technology Studies (STS)

**DOI:** 10.1017/cts.2025.10120

**Published:** 2025-09-01

**Authors:** Stephen Molldrem, Jacob D. Moses, Emma Tumilty, Peyton Swanson, Jeffrey S. Farroni, Elise M.R. Smith

**Affiliations:** 1 Institute for Bioethics and Health Humanities, The University of Texas Medical Branch, Galveston, USA; 2 Institute for Translational Sciences, The University of Texas Medical Branch, Galveston, USA

**Keywords:** Evaluation, Science and Technology Studies, Translational Science, CTSA evaluation, STS

## Introduction

One central goal of translational science is to develop innovations that positively impact clinical or population-level health and reduce disparities [1]. To track progress toward these goals, the Clinical and Translational Science Award (CTSA) program, the National Center for Advancing Translational Sciences (NCATS), and the translational science community have invested heavily in evaluating the field’s impact on individuals, scientific careers, biomedical innovation, and society [2,3]. Much progress has been made in evaluating the impact of translational science on specific outcomes, such as the success of CTSA training and pilot grants [4–6], returns on investment for particular innovations [7,8], and the impact of major initiatives like the Streamlined, Multi-site, Accelerated Resources for Trials (SMART) IRB platform [9]. However, measurable assessments of the field’s overall impact on biomedical innovation and society remain elusive, despite a desire for such metrics [10,11].

Using approaches from the field of Science and Technology Studies (STS), including archival and policy analysis methods, we have found that CTSA evaluators have regularly lamented falling short of aspirations to measure the impact of translational science on society, even while promoting the field’s potentially transformational impact [12]. By assessing key outcomes such as publications and grants received by CTSA-funded scientists and collaboration across hubs, NCATS has enacted what we call an “evaluative way,” whereby translational science’s self-assessment strategies have deeply shaped its innovations and raison d’être [12].

Against this backdrop, we argue that CTSA program stakeholders should incorporate STS scholars into the next generation of evaluation efforts to develop evaluation strategies suited to measuring the large-scale impacts of translational science. STS is an interdisciplinary field with a 50-plus year history of analyzing how scientific innovations reshape society – including describing how novel sciences develop, produce knowledge, disseminate innovations, and inform policy [13,14]. For example, STS scholars have studied the emergence of synthetic biology and nanotechnology since their inception, tracking these fields’ development and broader impacts on science and economic processes [15,16].

In mobilizing STS expertise to evaluate the CTSA program and translational science, NCATS can invest in examining the comparative historical, social, and policy-oriented drivers that have led major initiatives to succeed or fail. For example, undertaking long-term ethnographic research or historical studies using archival documents can potentially shed light on the larger-scale impacts of CTSA programming and translational initiatives more effectively than narrowly tailored evaluation metrics, therein opening new opportunities for improvement and innovation. This is partly because of the epistemological underpinnings of STS, which focus on the plurality of contingent ways in which knowledge is created, disseminated, and taken up. We now sketch out what it might look like to pursue these goals.

## STS and CTSA evaluation

STS scholars should become embedded in the CTSA network and its evaluation enterprise. STS uses varied methods to assess the development and impact of scientific disciplines – generally using embedded approaches such as long-term ethnographic fieldwork, interpretive policy analysis, qualitative methods, and archival research [14,17–19]. Like translational science, STS is not a discipline, but an interdisciplinary field with theoretical and applied aims, including to make science more responsive to social values. STS scholars can mobilize approaches to help ensure that future evaluation efforts are robustly informed by prior developments and are well-suited to meeting the CTSA program’s desire to understand translational science’s impact on science and society [1,12,19]. Luckily, STS scholars are already present at most universities across departments and disciplines, constituting readymade potential partners and collaborators for CTSAs.

Translational science is not entirely foreign to STS. However, to date, STS scholars have studied translational science as a research object, without attempting to develop applied tools that could contribute to the practice of the science [20]. This work has documented the impacts of translational science on the health sciences and society, including on “translational lag” discourses [21], the emergence of new public-private partnership models [22,23], and the role of evaluation in the emergence of translational science [12]. We suggest that STS scholars not only pursue studies *of* translational science, but also become embedded *within* translational enterprises and CTSA hubs. Supporting STS research can help fulfill longstanding goals within translational science to measure the field’s large-scale impacts, because STS provides methods for studying how translational science has sought to transform how biomedical knowledge is produced and disseminated. Therefore, being embedded in CTSAs can allow STS scholars to identify internal factors that shape translational innovation while also foregrounding STS’s broader focus of tracking the social, political, and economic impacts of scientific discovery.

Table 1 shows “enduring areas of evaluative focus” and “example measurements” in CTSA program evaluation history, drawn from a paper by our group [12]. It then presents potential STS methods that could be mobilized to evaluate translational science and its impacts on science and society across a variety of scales.

**Table 1.** Existing models and potential STS approaches for evaluating the impact of the CTSA program

Area of Evaluative Focus in the CTSA Program (from [12])	Example Measurements from CTSA Program History (from [12])	Potential STS Evaluation Methods
Overall impact of translational science on biomedical innovation	The three “Common Metrics” used from 2015-2022: Careers in clinical and translational research Institutional review board duration; and Pilot funding and publications	Historical STS methods, including archival research and document analysis, to trace large-scale changes in the relationship between science and society to identify and describe major impacts of the CTSA program on key developments
Scientific careers in translational research	Number of graduates from translational science programs Counts of published works R01 granted to graduates from translational science programs	Ethnographic research following translational scientists’ careers from graduate training onward to assess the translational impact of their work Document analysis and ethnographic research to trace the impact of specific papers on translational innovations
Research productivity among translational researchers	Number of pilots granted Number of publications resulting from pilots Number of R01s granted to individuals that were granted a pilot Funding and publications/research productivity	Using published bibliometric and CTSA evaluation studies to identify impactful translational teams to engage in long-term qualitative STS research, with the aim of understanding drivers of impact Ethnographic research with funders to understand perspectives on translational innovation
Lessening regulatory burden for conducting translational research	Time between when a study protocol is sent to the IRB and accepted Subject accrual and retention pilot	Multi-sited ethnographic research with regulatory professionals at IRBs and federal agencies to understand translational science barriers Comparative case studies of approval processes with different outcomes Cross-national comparison of translational science policy initiatives to bring regulatory lessons from other countries or regions to the US
Collaboration to support translational research	Number of internal and external partnerships Individuals writing papers together Individuals writing grants together Success of translational teams Big data-focused collaborations Support for Team Science and the Science of Team Science	Multi-sited ethnographic research with collaborative translational teams to understand comparative success Interview studies with highly successful and unsuccessful teams to understand barriers, facilitators, and impact on fields
Community engagement	Number of community engagement events Number of individuals attending events Number of community members trained in research evaluation and added to community expert pool Number of translational teams engaging in community engagement activities Number of community-based pilot grants applications (including number of new community-campus partnerships, and number of submissions) Number of TL1 pre/post-doc trainees taking part in community engagement training	Ethnographic research with translational teams and communities they engage, to assess differing perceptions of impact and knowledge generation Archival and ethnographic research with patient associations and civil society organizations that conduct advocacy around translational innovation Long-term participant-observation fieldwork across CTSA community engagement cores, to understand the knowledge they produce and impact on scientific practices and society Ethnographic research with national CTSA community engagement groups such as Partners for the Advancement of Community Engaged Research (PACER)
Demographic diversity in the translational science workforce	Number of underrepresented graduates/researchers in NIH T and K Award programs Number of women in CTSA faculty positions Number of underrepresented minorities in ITS/CTSA faculty positions Equity Evaluation Feedback Diversity, Equity, Inclusion, and Accessibility (DEIA) Metrics	Use of frameworks such as racialization and feminist STS to study the experience of being a minoritized person in translational science, power dynamics, and how these shape one’s ability to impact the field and the creation of translational knowledge that can reshape society [24,25] Studies of how metrics related to diversity are created, used in practice, or abandoned
Social and economic impact of translational science	Very few until the recent history of the CTSA program (see, [11]). See also, [1]	Mixed methods analyses of policy documents and federal economic datasets to assess the impact of translational science on the knowledge economy
Unified metric (or “meta-metric”) of translational science initiatives and impact	Seen as the great challenge of evaluating translational science Numerous attempts, including the Common Metrics Initiative and Translational Science Benefits Model (TSBM, see, [11])	Creating STS models to study the impacts of translational science projects at different scales by focusing on the process of knowledge-creation within initiatives and then tracing how outputs impacted areas of biomedicine and society

Models for embedding diverse disciplines exist in the CTSA program in community engagement, biostatistics, research ethics, dissemination and implementation (D&I), team science, and evaluation. STS scholars can partner with these formations while also becoming embedded in translational initiatives as autonomous experts. Our CTSA is one site where STS is becoming embedded, facilitated through our longstanding embedded ethics program, where humanities faculty engage in CTSA activities related to ethics, team science, community engagement, D&I, and other areas. In addition to embedding STS in CTSAs, NCATS should develop mechanisms to directly fund STS scholars to conduct large-scale studies of translational impact. This work can also contribute to the further development of STS literatures.

## Conclusion

NCATS has consistently framed measuring translational science’s impact on biomedical innovation and society as a central goal. However, even after efforts such as the Common Metrics Initiative, the field still falls short of this [10–12]. STS offers methods well-suited to assessing the impact of translational science on the health sciences and society-at-large that operate at different registers than traditional evaluation [12,20–23]. By working collaboratively with existing CTSA evaluation efforts while bringing new intellectual resources to bear on evaluating translational science, STS can advance the field while enriching understandings of how translational ways of knowing are remaking the world.
